# Potential Malaria Reemergence, Northeastern Thailand

**DOI:** 10.3201/eid1508.090240

**Published:** 2009-08

**Authors:** Trevor Petney, Paiboon Sithithaworn, Rojchai Satrawaha, Carl Grundy-Warr, Ross Andrews, Yi-Chen Wang, Chen-Chieh Feng

**Affiliations:** University of Karlsruhe, Karlsruhe, Germany (T. Petney); Khon Kaen University, Khon Kaen, Thailand (P. Sithithaworn); Mahasarakham University, Mahasarakham, Thailand (R. Satrawaha); National University of Singapore, Singapore (C. Grundy-Warr, Y.-C. Wang, C.-C. Feng); University of South Australia, Adelaide, South Australia, Australia (R. Andrews)

**Keywords:** Thailand, malaria, *Anopheles dirus*, rubber plantations, land use change, susceptible population, parasites, letter

**To the Editor:** The emergence and reemergence of infectious diseases are major problems for healthcare systems worldwide. Unfortunately, because accurate prediction of the occurrence of such diseases is difficult, if not impossible, surveillance and control can be carried out only after the outbreak has occurred. Predicting the likelihood of a disease outbreak should make it possible to start surveillance programs before outbreaks occur and to initiate control programs before the population has become seriously affected. We used data on changes in land use patterns to predict the likelihood of malaria reemergence in northeastern Thailand.

Because natural rubber is of major economic importance and cannot be replaced by synthetic alternatives, the demand for and production of this commodity has consistently increased (*1*). This situation has led to changes in agricultural practices in various countries in Southeast Asia; rubber production has increased in Myanmar, Laos, Thailand, and Vietnam (*1*,*2*).

Northeastern Thailand (Isaan) is a relatively poor area, and most rubber plantations belong to smallholders and provide them with a large source of income (*3*). In 1993, ≈284 km^2^ of northeastern Thailand were covered by rubber plantations; this area increased to 422 km^2^ in 1998 and to 948 km^2^ by 2003 (*3*). Since then, planting has increased exponentially so that, by 2006, the total area planted with rubber was >2,463 km^2^; new plantings expanded another ≈1,345 km^2^ from 2004 to 2006 and increased to a total of 5,029 km^2^ in 2007 (*3*). The plants mature ≈6 years after planting; at that stage, the trees can reach 10–12 m in height, although the growth rate depends on the physical and biotic environment (*4*).

Deforestation in northeastern Thailand early in the last century led to an extreme reduction in the incidence of malaria (*5*) because the main vector mosquito in this area, *Anopheles dirus* sensu stricto, is forest dwelling and requires a shaded environment for its survival and reproduction (*6*). Currently, the northeastern part of the country is relatively free of autochthonous malaria cases except for 3 provinces that border Cambodia and Laos (*5*), Srisaket, Ubon Ratchathani, and Surin. In Srisaket and Ubon Ratchathani, 25% and 31%, respectively, of malaria cases are imported, particularly from Cambodia (*7*).

Mosquitoes are sensitive to changes in environmental conditions, such as shade, temperature, and humidity. These conditions are often influenced by land use change, such as conversion of rice paddies to rubber plantations (*8*). In addition to providing economic benefits for the population, rubber plantations also provide suitable habitats for *A. dirus* s.s., perhaps even better habitats than those found in the original rain forest; new plantations lead to increased mosquito density and disease incidence (*8*). Thus, planting large tracts of rubber potentially increases the likelihood of the reemergence of malaria in northeastern Thailand, although a malaria vector such as *A. dirus* s.s. could return without reemergence of the disease (*9*).

Should malaria return, the greatly reduced contact between the local Isaan population and *Plasmodium* spp. over the past ≈50 years suggests that malaria would enter a highly susceptible population, potentially leading to major health problems at the individual and regional levels. This possibility is of particular concern because several strains of *Plasmodium* in Thailand and surrounding countries are multidrug resistant, which leads to treatment difficulties (*5*).

Each land use change creates different microclimatic conditions, which directly and indirectly affect the occurrence and distribution of malaria (*10*). Whether malaria will return as a major health threat likely depends on the size and fragmentation of the individual plantation areas. The required size of a plantation for the survival of the vector population is unclear, but large areas of plantation tend to offer dense vegetation and, therefore, high humidity and shade, which provide suitable environmental conditions for larval habitats, even during the dry season (*8*). Conversely, during the rainy season, conditions at the edges of fragmented forests, where human settlements are often located, become favorable for larval habitats, rendering villagers susceptible to the disease (*6*). In addition to changes in habitat and microclimate, social or political changes in the region may affect the transborder movement of malaria into Thailand with consequences for potential reemergence (*7*).

Although the association between rubber plantations and malaria is well known in Southeast Asia, the potential for reemergence should receive substantially more attention from economic, agricultural, and environmental planning bodies. Changes in land use and land cover have the potential to facilitate the transmission of disease to humans. Understanding the influence of land use change on malaria occurrence is critical for shaping future surveillance and control strategies.

## References

[1] van Beilen JB, Poirier Y. Establishment of new crops for the production of natural rubber. Trends Biotechnol. 2007;25:522–9. 17936926 10.1016/j.tibtech.2007.08.009

[2] Food and Agriculture Organization. Selected indicators of food and agriculture development in Asia Pacific Region 1992–2002. Bangkok: The Organization; 2003.

[3] Rubber Research Institute of Thailand [in Thai]. [cited 2009 Jun 24]. Available from http://www.rubberthai.com

[4] Wright H. *Hevea brasiliensis,* or para rubber, its botany, cultivation, chemistry and diseases, 2nd ed. Colombo (Sri Lanka): A.M. & J. Ferguson; 1906.

[5] Chareonviriyaphap T, Bangs MJ, Ratanatham S. Status of malaria in Thailand. Southeast Asian J Trop Med Public Health. 2000;31:225–37. 11127318

[6] Obsomer V, Defourny P, Coosemans M. The *Anopheles dirus* complex: spatial distribution and environmental drivers. Malar J. 2007;6:26. 10.1186/1475-2875-6-26 17341297 PMC1838916

[7] Thimasarn K, Jatapadma S, Vijaykadga S, Sirichaisinthop J, Wongsrichanalai C. Epidemiology of malaria in Thailand. J Travel Med. 1995;2:59–65. 10.1111/j.1708-8305.1995.tb00627.x 9815363

[8] Yasuoka J, Levins R. Impact of deforestation and agricultural development on anopheline ecology and malaria epidemiology. Am J Trop Med Hyg. 2007;76:450–60. 17360867

[9] Lindblade KA, Walker ED, Onapa AW, Katungu J, Wilson ML. Land use change alters malaria transmission parameters by modifying temperature in a highland area of Uganda. Trop Med Int Health. 2000;5:263–74. 10.1046/j.1365-3156.2000.00551.x 10810021

[10] Fantini B. Anophelism without malaria: an ecological and epidemiological puzzle. Parassitologia. 1994;36:83–106. 7898963

